# Immune-mediated diseases are associated with a higher incidence of dementia: a prospective cohort study of 375,894 individuals

**DOI:** 10.1186/s13195-022-01072-x

**Published:** 2022-09-13

**Authors:** Ya-Ru Zhang, Liu Yang, Hui-Fu Wang, Bang-Sheng Wu, Shu-Yi Huang, Wei Cheng, Jian-Feng Feng, Jin-Tai Yu

**Affiliations:** 1 Department of Neurology and Institute of Neurology, Huashan Hospital, State Key Laboratory of Medical Neurobiology and MOE Frontiers Center for Brain Science, Shanghai Medical College, Fudan University, National Center for Neurological Disorders, 12th Wulumuqi Zhong Road, Shanghai, 200040 China; 2 Institute of Science and Technology for Brain-Inspired Intelligence, Fudan University, Shanghai, China; 3 Key Laboratory of Computational Neuroscience and Brain-Inspired Intelligence (Ministry of Education), Fudan University, Shanghai, China; 4 Fudan ISTBI—ZJNU Algorithm Centre for Brain-Inspired Intelligence, Zhejiang Normal University, Jinhua, China; 5 MOE Frontiers Center for Brain Science, Fudan University, Shanghai, China; 6 Zhangjiang Fudan International Innovation Center, Fudan University, Shanghai, China

**Keywords:** Dementia, Alzheimer’s disease, Immune-mediated diseases, Neutrophils, Lymphocytes

## Abstract

**Background:**

Immune system dysregulation plays a vital role in the pathogenesis of neurodegenerative diseases, even considered to be as important as classical pathological protein aggregation assumption. However, the associations of immune-mediated diseases with incident dementia are unclear and need to be clarified in prospective studies with a large population and long follow-up time.

**Methods:**

We investigated the relationship between any or individual immune-mediated diseases and incident dementia based on a prospective cohort UK Biobank. The risk for dementia was assessed with multivariable hazard ratio (HR) and 95% confidence interval (CI) after adjusting for various potential confounders using time-varying Cox proportional hazards regression. We also performed the subgroup Cox analysis stratified by time since immune-mediated diseases and gender. Causal mediation analyses with 1000 bootstrapped iterations were conducted to explore the mediation effects of peripheral immune cells on the associations of immune-mediated diseases with dementia.

**Results:**

A total of 375,894 participants were included in the study, among which 5291 developed dementia during a median follow-up of 9.08 years. Immune-mediated diseases were associated with an increased risk of dementia (HR, 1.10; 95% CI, 1.00–1.21), and the risk was highest between 1 and 2 years after immune-mediated diseases onset (HR, 2.74; 95% CI, 1.86–4.04). Females who suffered from immune-mediated diseases were more prone to AD, while males were more susceptible to VD. Four of the individual immune-mediated diseases including type I diabetes mellitus (HR, 2.49; 95% CI, 1.97–3.15), rheumatic fever or rheumatic heart diseases (HR, 1.36; 95% CI, 1.05–1.77), multiple sclerosis (HR, 2.87; 95% CI, 1.92–4.30), and necrotizing vasculopathies (HR, 1.71; 95%CI, 1.03–2.85) were significantly related to higher dementia incidence. The relationship between immune-mediated diseases and dementia was partially mediated by peripheral immune cells including neutrophils and lymphocytes.

**Conclusions:**

In this large cohort study, immune-mediated diseases were proven to be significantly associated with an increased risk of incident dementia, especially for type I diabetes mellitus which was observed to be related to the higher incidence of all types of dementia. Our findings could provide new sights on dementia pathogenesis and intervention from the perspective of systemic immunology and immune-mediated diseases.

**Supplementary Information:**

The online version contains supplementary material available at 10.1186/s13195-022-01072-x.

## Introduction

Aging accompanied neurodegeneration involving neuronal dysfunction and loss is critical in dementia pathogenesis. Since Alzheimer’s disease (AD) was first characterized by Alois Alzheimer in 1907, who found the hallmarks of extracellular plaques and intracellular neurofibrillary tangles, the mechanism studies of dementia in the past century mainly focused on protein aggregation such as amyloid β (Aβ), tau, α-synuclein, and transactive response DNA-binding protein 43 kDa (TDP-43) [1, 2]. In addition to protein aggregation, there has been growing recognition regarding the role of immune system dysfunction in neurodegenerative pathogenesis. Immune signaling molecules including the transforming growth factors, the interleukins, the complements, and the triggering receptor expressed on myeloid cells 2 (TREM2) directly cause neuron damage or indirectly interact with the protein aggregation process, which contributes to neurodegeneration [3–5]. Immune cells, including neutrophils and lymphocytes, were also proven to influence the neurodegenerative pathology [6, 7] and modify the risk of dementia [8, 9]. Corresponding immune-modulatory agents for dementia, especially AD, were set into preclinical or clinical investigation, although the efficiency is still controversial [10].

Immune-mediated diseases are systemic diseases of complex, multifactorial etiology in the context of immune system dysfunction as well, including but not limited to inflammatory bowel diseases, multiple sclerosis, type I diabetes, systemic lupus erythematosus, and rheumatoid arthritis [11, 12]. There have been studies revealing genetic links between immune-mediated diseases and dementia. One study found fifteen overlapped single-nucleotide polymorphisms (SNPs) within frontotemporal dementia (FTD) and immune-mediated diseases, and over half of the SNPs were mapped to the human leukocyte antigen (HLA) region, which is an area rich in genes related to microglial function [13]. Another study identified eight overlapped SNPs associated with the risk of both AD and immune-mediated diseases [14], and one of the loci was in the HLA region too. Further, the aforementioned immune molecules and cells in neurodegeneration like interleukins, neutrophils, and lymphocytes were also implicated as critical contributors in immune-mediated diseases [15]. These findings supported that immune-mediated diseases shared similar genetic, molecular, and cellular pathways with dementia, which may predict the onset of dementia.

To fully unravel the associations of immune-mediated diseases with incident dementia, we investigated the risk for dementia in immune-mediated diseases patients compared to healthy controls based on a prospective cohort UK Biobank (UKB) taking advantage of long follow-up time and large population. Considering the critical role of peripheral immune cells in both immune-mediated diseases and dementia, we further performed the causal mediation analyses to explore the effects of peripheral neutrophils and lymphocytes on the associations of immune-mediated diseases with dementia.

## Methods

### Study participants

The participants and data resources involved in this study were from a population-based prospective cohort UKB, under application number 19542. Between 2006 and 2010, over 500,000 participants aged 40–69 years were recruited in UKB with a comprehensive baseline assessment of health status at 22 centers throughout the UK [16]. Follow-up is conducted chiefly through linkages to routinely available national datasets, including death register data, cancer register data, hospital inpatient data, and primary care data (https://www.ukbiobank.ac.uk/). Environmental, genetic, disease status, and other clinical data were obtained from questionnaires, physical measures, sample assays, and linked electronic health data. Ethics approval for the UKB study was obtained from the North West Multicenter Research Ethical Committee. All participants provided written informed-consent paper at baseline. We restricted analyses to individuals with hospital inpatient data where the diagnosis information based on International Classification of Diseases (ICD)-10 codes were available to identify immune-mediated diseases patients. The control comparators were defined as the individuals with hospital inpatient data but none of the immune-mediated diseases was diagnosed. Prevalent dementia patients at baseline and those without traceable outcome data were excluded.

### Ascertainment of the exposure variable immune-mediated disease

We identified immune-mediated diseases' status and diagnosis date by matching the corresponding ICD-10 codes [17] from hospital inpatient data (HESIN). If two or more different diagnosis dates were recorded for the same disease, the earliest one was used as the primary diagnosis date. Only the diseases with at least 500 affected individuals were kept for further analyses because the dementia incidence was low in some rare immune-mediated diseases. A total of 20 immune-mediated diseases were ascertained (Supplementary Table 1). We assessed the longitudinal associations of any immune-mediated diseases and the individual immune-mediated diseases with dementia. The immune-mediated diseases were required to be diagnosed at least one year before the dementia diagnosis.

### Ascertainment of the outcome variable incident dementia

Incident all-cause dementia (ACD), AD, and vascular dementia (VD) were ascertained and classified according to the ICD codes and Read codes (Supplementary Table 2). ACD covered all the types of dementia including AD, VD, FTD, dementia with Lewy bodies (DLB), Parkinson’s disease dementia (PDD), dementia in corticobasal degeneration (CBD), dementia in other neurodegenerative, and specified diseases. The outcome records were extracted from algorithmically defined (Fields 42,018–42,025), first occurrences data reported (Fields 131,036–131,037, 130,836–130,843), death register data documented (Fields 40,001–40,002), HESIN summarized (Fields 41,270–41,271, 41,280–41,281), and primary care data recorded (Field 42,040) dementia. Follow-up visits began from the date of attending the assessment center (Field 53) to the earliest incident dementia diagnosis, date of death, the last data collection date by the general practitioner, or the last time of hospital inpatient admission, whichever occurred first.

### Ascertainment of the mediator variable peripheral immune cell

Blood samples collected in ethylenediaminetetraacetic acid (EDTA) vacutainers of the UKB participants were analyzed at the UKB central laboratory within 24 h of blood draw. Differential leukocytes counts were acquired from the automated quantitative analyzer, the Beckman Coulter LH750 Hematology Analyzer. We extracted the blood count data of neutrophils and lymphocytes collected after immune-mediated diseases diagnosis and before dementia onset.

### Statistical analyses

Baseline characteristics of participants were summarized for those with and without incident dementia status as mean and standard deviation (SD) for continuous variables, number, and percentage for categorical variables. Longitudinal associations of immune-mediated diseases with incident dementia were examined with multivariable time-varying Cox proportional hazard regression models and presented with hazard ratio (HR) and 95% confidence interval (CI). Participants who died, withdrew from the study before the end of follow-up, or had not developed dementia by the end of follow-up (April 7, 2021) were censored. In the primary analysis, we performed the model unadjusted, then adjusted for age, sex, *ApoE-ε4*, education, ethnicity, body mass index (BMI), socioeconomic status indicated by Townsend deprivation score, smoking, and alcohol consumption. The *p* values were further adjusted to control the false discovery rate (FDR) at 5% using the Benjamini–Hochberg procedure (labeled as *Q* values) when analyzing the associations of 20 individual immune-mediated diseases with dementia. In secondary analyses, we explored the effect of time since immune-mediated diseases on dementia. Time since immune-mediated diseases was split into overlapping periods (1 to 2 years, 1 to 3 years, 1 to 4 years, 1 to 5 years, 1 to 10 years, 1 to 15 years, 1 to 20 years, and 1 to over 20 years). We also estimated the effect differences of immune-mediated diseases on dementia across sex groups at recruitment. To evaluate the potential reverse causation biases, we also performed the sensitivity analyses by excluding the participants with follow-up time less than 5 or 10 years as suggested in work conducted by Sipila et al. [18]. Finally, the mediation analyses based on the methods proposed by Baron and Kenny [19] with the significance determined using 10,000 bootstrapped iterations were conducted to investigate whether the associations of immune-mediated diseases with dementia were mediated by peripheral immune cells which were proven to be related to dementia incidence in our previous study based on UKB. In addition, the interaction analyses were performed between sex and peripheral immune cells in contributing to the dementia incidence.

All the above analyses were carried out using R version 4.0.2. A *p*-value of less than 0.05 was considered to be statistically significant.

## Results

### Population characteristics

Among 502,493 participants at baseline in the UKB, 375,894 participants with HESIN ICD-10 data were eligible for Cox analysis after excluding 888 participants with prevalent dementia at baseline and 33,527 participants without follow-up data (Supplementary Fig. 1). Baseline characteristics of the included participants are presented by incident dementia status (Table 1) and immune-mediated diseases status (Supplementary Table 3). Overall, the mean age of participants was 57.32 (± 7.99) years, and 205,575 (54.69%) of them were females. Among these participants, 58,589 were diagnosed with at least one of the 20 immune-mediated diseases 1 year before dementia onset. During a median follow-up of 9.08 years, 5291 participants developed dementia, of which 2301 cases were AD and 1249 cases were VD.

**Table 1:** Baseline characteristics of UKB participants by dementia status (*N* = 375,894)

	No incident dementia (*N* = 370,603)	Incident ACD (*N* = 5291)	Incident AD (*N* = 2301)	Incident VD (*N* = 1249)
Age, years	57.22 (7.98)	64.35 (4.75)	64.75 (4.21)	64.81 (4.27)
Female, *n* (%)	203,123 (54.81)	2452 (46.34)	1186 (51.54)	501 (40.11)
*ApoE ε4* carriers, *n* (%)	89,731 (24.21)	2374 (44.87)	1240 (53.89)	525 (42.03)
Higher education, *n* (%)	130,207 (35.13)	1336 (25.25)	555 (24.12)	260 (20.82)
Ethnicity_White, *n* (%)	349,469 (94.30)	5003 (94.56)	2199 (95.57)	1179 (94.40)
BMI, kg/m^2^	27.69 (4.91)	27.92 (5.05)	27.40 (4.77)	28.68 (5.23)
Townsend deprivation score	-1.24 (3.12)	-0.82 (3.37)	-1.02 (3.28)	-0.66 (3.46)
Smoking				
Never, *n* (%)	194,964 (52.61)	2363 (44.66)	1080 (46.94)	505 (40.43)
Previous, *n* (%)	132,625 (35.79)	2266 (42.83)	979 (42.55)	572 (45.80)
Current, *n* (%)	40,753 (11.00)	597 (11.28)	213 (9.26)	152 (12.17)
Alcohol consumption				
Never, *n* (%)	17,008 (4.59)	382 (7.22)	177 (7.69)	85 (6.81)
Previous, *n* (%)	14,526 (3.92)	399 (7.54)	149 (6.48)	118 (9.45)
Current, *n* (%)	337,832 (91.16)	4471 (84.50)	1960 (85.18)	1037 (83.03)
Immune-mediated diseases				
None, *n* (%)	313,216 (84.52)	4089 (77.28)	1835 (79.75)	909 (72.78)
Any, *n* (%)	57,387 (15.48)	1202 (22.72)	466 (20.25)	340 (27.22)

Data presented as mean (SD) for continuous variables and number (%) for categorical variables. Higher education refers to college/university/other professional qualification

*ACD* All-cause dementia, *AD* Alzheimer’s disease, *VD* Vascular dementia, *BMI* Body mass index

### Any immune-mediated diseases and dementia risk

**Fig. 1:**
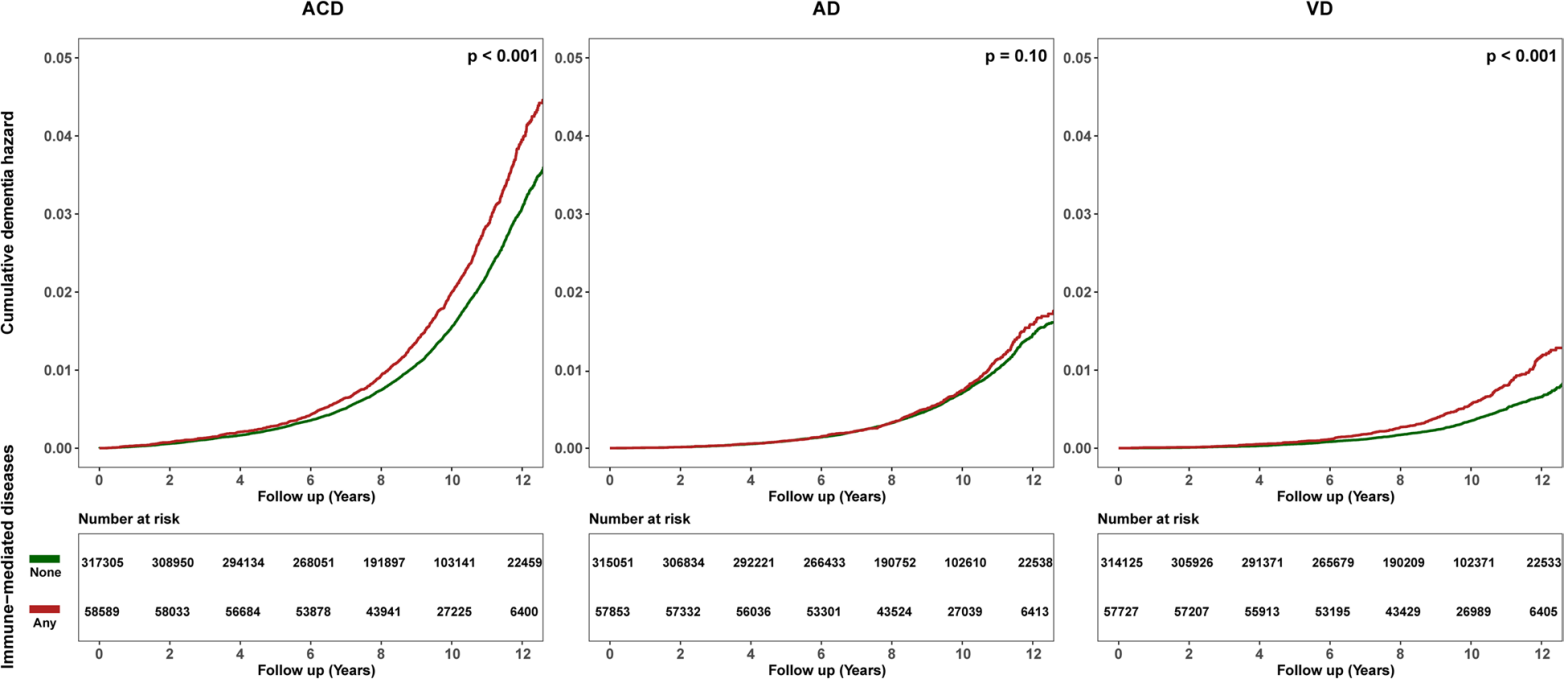
The cumulative dementia hazard according to the status of immune-mediated diseases. ACD, all-cause dementia; AD, Alzheimer’s disease; VD, vascular dementia

**Fig. 2:**
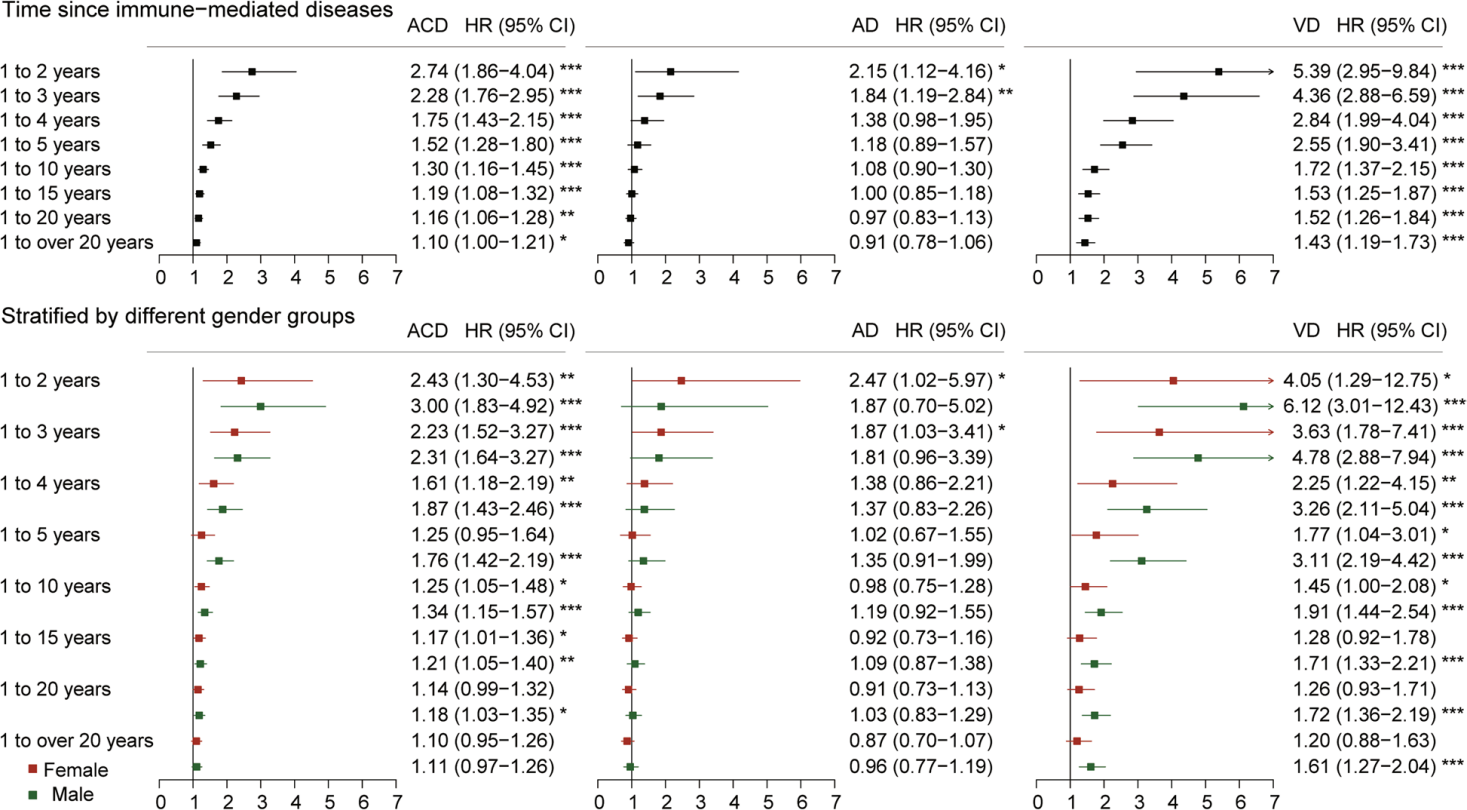
Risk for incident dementia according to the status of immune-mediated diseases stratified by time since immune-mediated diseases. Time periods are overlapping. Adjusted for age, sex, education, *ApoE-ε4*, ethnicity, BMI, Townsend deprivation score, smoking and alcohol consumption (^*^p < 0.05, ^**^p < 0.01, ^***^p < 0.001). ACD, all-cause dementia; AD, Alzheimer’s disease; VD, vascular dementia; HR, hazard ratio; CI, confidence interval

Immune-mediated diseases in whole were associated with increased risk of ACD (HR, 1.10; 95% CI, 1.00–1.21) and VD (HR, 1.43; 95% CI, 1.19–1.73) while not associated with AD after multivariable adjustment for age, sex, education, *ApoE-ε4*, ethnicity, BMI, Townsend deprivation score, smoking, and alcohol consumption (Fig. 1 and Supplementary Table 4). When split the time since immune-mediated diseases into overlapping periods, the results were still statistically significant for ACD and VD in all periods. A similar risk efficacy was also observed for AD in three years before immune-mediated diseases diagnosis (Fig. 2). The risk of dementia was highest between 1 and 2 years after immune-mediated diseases onset for ACD (HR, 2.74; 95% CI, 1.86–4.04), AD (HR, 2.15; 95% CI, 1.12–4.16), and VD (HR, 5.39; 95% CI, 2.95–9.84). Subgroup analyses of sex stratification indicated that females with immune-mediated diseases were more prone to AD in the early years after immune-mediated disease (Fig. 2), while males were more susceptible to VD during a long follow-up time period (Fig. 2 and Supplementary Table 4). Sensitivity analyses showed that the risk associations with ACD and VD remained when immune-mediated diseases occurred more than 5 years before the diagnosis of dementia (Supplementary Table 5). While when dementia cases occurred from 10 years onward, the associations for ACD only persisted in models unadjusted or adjusted for age, sex, *ApoE-ε4*, and education, and the associations for VD were consistently robust in all models (Supplementary Table 5).

### Individual immune-mediated diseases and dementia risk

**Fig. 3:**
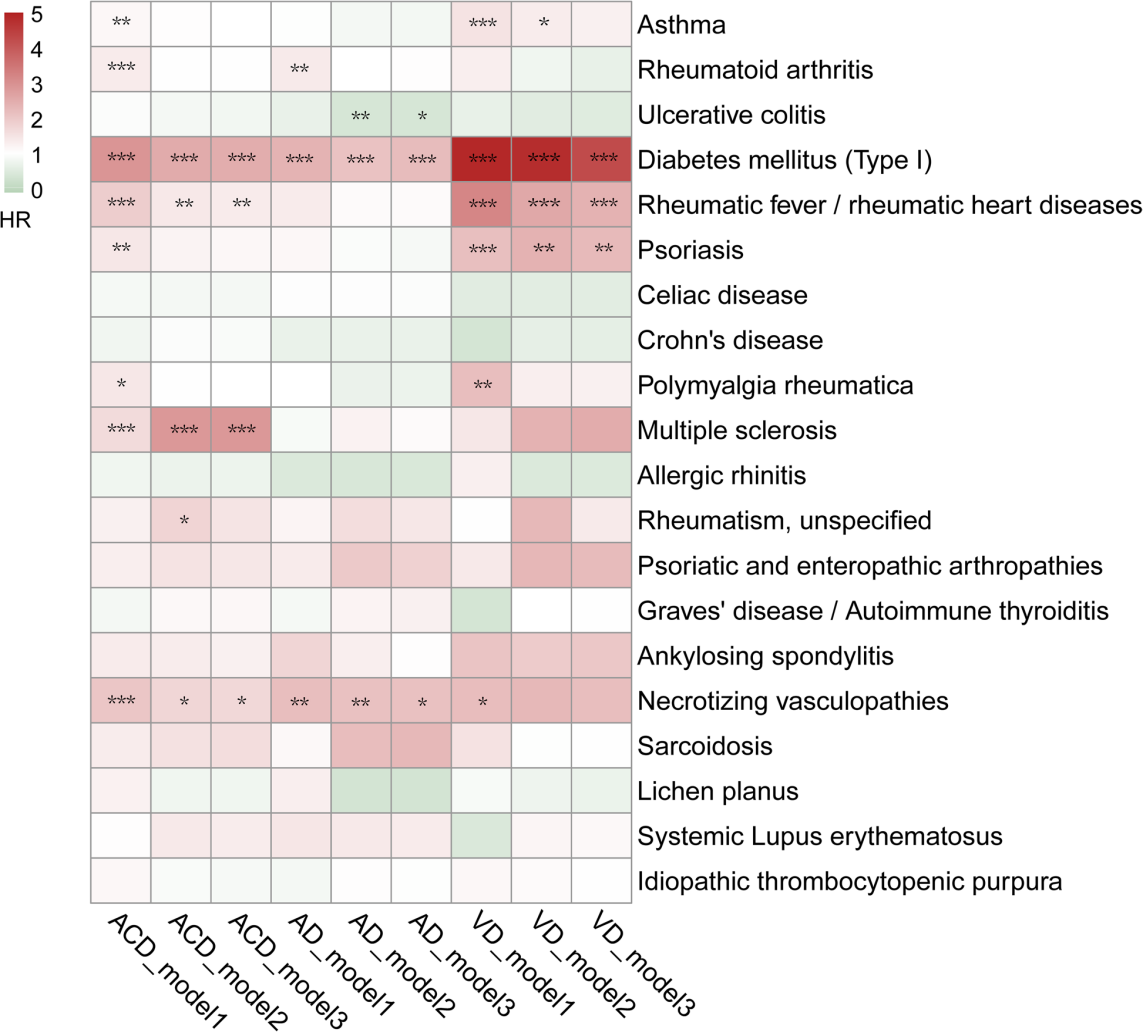
Risk for incident dementia according to the status of individual immune-mediated diseases. Model 1 unadjusted. Model 2 adjusted for age, sex, *ApoE-ε4*, and education. Model 3 adjusted for age, sex, education, *ApoE-ε4*, ethnicity, BMI, Townsend deprivation score, smoking, and alcohol consumption (^*^p < 0.05, ^**^p < 0.01, ^***^p < 0.001). ACD, all-cause dementia; AD, Alzheimer’s disease; VD, vascular dementia; HR, hazard ratio

For individual immune-mediated diseases, participants were at higher risk of developing ACD when suffering from type I diabetes mellitus (HR, 2.49; 95% CI, 1.97–3.15), rheumatic fever or rheumatic heart diseases (HR, 1.36; 95% CI, 1.05–1.77), multiple sclerosis (HR, 2.87; 95% CI, 1.92–4.30), and necrotizing vasculopathies (HR, 1.71; 95%CI, 1.03–2.85) (Fig. 3 and Supplementary Table 6). However, the effect on subtypes of dementia varies in different immune-mediated diseases. Type I diabetes mellitus was the only disease consistently related to a higher risk of both AD (HR, 2.21; 95% CI, 1.49–3.28) and VD (HR, 4.25; 95% CI, 2.90–6.23). Necrotizing vasculopathies were related to a higher incidence of AD (HR, 2.13; 95% CI, 1.06–4.27), while ulcerative colitis was related to a lower incidence of AD (HR, 0.37; 95% CI, 0.17–0.83). Rheumatic fever or rheumatic heart diseases (HR, 1.36; 95% CI, 1.05–1.77) and psoriasis (HR, 1.36; 95% CI, 1.05–1.77) were proven to be risk factors for VD. In further FDR adjusting, the *Q* values remained statistically significant for associations of type I diabetes mellitus with ACD, AD and VD, for multiple sclerosis with ACD, for rheumatic fever or rheumatic heart diseases and psoriasis with VD (Supplementary Table 6). Sensitivity analyses showed that the results remained significant for associations of ACD with type I diabetes mellitus and multiple sclerosis, VD with type I diabetes mellitus and rheumatic fever or rheumatic heart diseases when immune-mediated diseases occurred more than 5 or 10 years before the diagnosis of dementia (Supplementary Table 7).

### Peripheral immune cells mediated associations of immune-mediated diseases with dementia

**Fig. 4:**
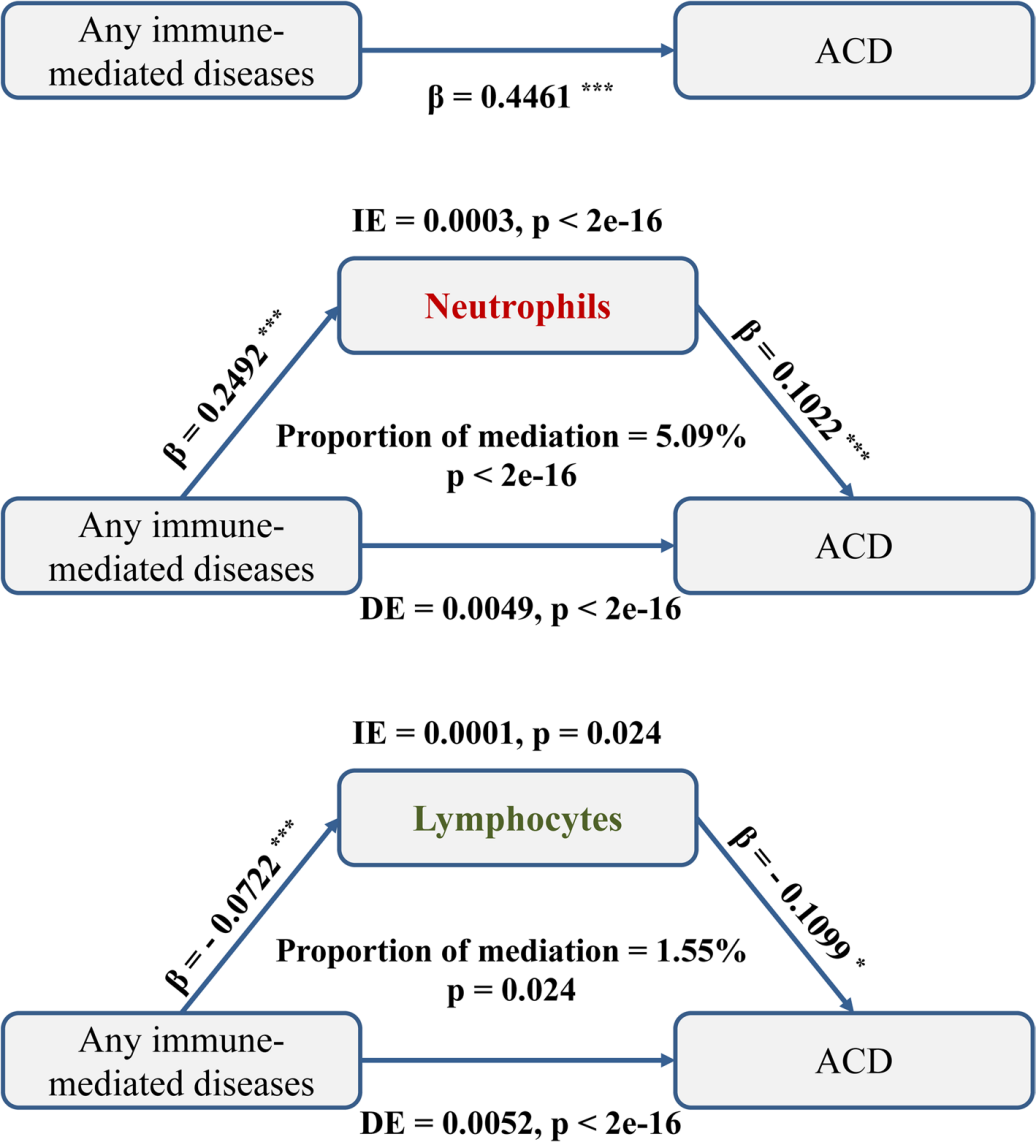
Peripheral immune cells mediation models of the associations between immune-mediated diseases and dementia. Controlling for age, sex, education, *ApoE-ε4*, ethnicity, BMI, Townsend deprivation score, smoking, and alcohol consumption (^*^p < 0.05, ^**^p < 0.01, ^***^p < 0.001). ACD, all-cause dementia; IE, indirect effect; DE, direct effect

Mediation analyses revealed that the associations between any immune-mediated diseases and dementia was partially mediated by peripheral immune cells, including neutrophils with the proportion of mediation 5.09% (*p* < 0.001) and lymphocytes with the proportion of mediation 1.55% (*p* = 0.024) respectively (Fig. 4). As for individual immune-mediated diseases, the risk effects on dementia of type I diabetes mellitus (Supplementary Fig. 2A), multiple sclerosis (Supplementary Fig. 2B), rheumatic fever or rheumatic heart diseases (Supplementary Fig. 2C), and necrotizing vasculopathies (Supplementary Fig. 2D) were partially mediated by neutrophils while not by lymphocytes. What is more, the interaction analyses results indicated that there was an interaction between sex and peripheral immune cells in contributing to the dementia incidence (Supplementary Table 8).

## Discussion

This large population-based study found that immune-mediated diseases were associated with an increased risk of dementia after adjusting for potential confounders. Among these diseases, type I diabetes mellitus was the only one promoting the higher incidence of ACD and the main subtypes of dementia. The risk of dementia was significantly high between 1 and 3 years following immune-mediated diseases onset, which might be due to reverse causality that undiagnosed dementia may increase the risk of immune-mediated diseases. Nevertheless, such associations persisted for more than 20 years that the relationships were still robust for ACD and VD. Notably, apparent sex differences were observed that females with immune-mediated diseases were more prone to AD in the early years after immune-mediated disease, while males were more susceptible to VD during a long follow-up time period. Finally, the mediation analyses revealed that the associations were partially mediated by peripheral immune cells neutrophils and lymphocytes.

To our knowledge, the current study is the most comprehensive and largest cohort study to investigate the effect of various immune-mediated diseases on dementia risk compared with previous studies. Our findings of type I diabetes mellitus are highly consistent with prior retrospective [20] and prospective [21] cohort studies which verified its risk effect on dementia even stronger than type II diabetes. Further studies indicated that poor blood glucose control [22] or severe hypoglycemic and hyperglycemic events [23] would worsen the condition. The potential mechanisms may be attributed to type I diabetes associated dementia biomarkers alterations [24] and brain atrophy [25]. As for rheumatic diseases, several retrospective studies [26–29] explored the impact of rheumatoid arthritis, Sjögren’s syndrome, and systemic lupus erythematosus on dementia, but the results were too heterogeneous to come to reliable conclusions, while none studies about rheumatic fever or rheumatic heart diseases were retrieved. The associations of multiple sclerosis and necrotizing vasculopathies with dementia risk have never been discussed. Recently, some researchers examined the associations between inflammatory bowel diseases and dementia. One of the studies utilizing the data from UKB did not find significant associations between inflammatory bowel diseases and dementia [30], which is in line with our findings. However, the other two studies reported a higher risk of dementia among inflammatory bowel diseases patients [31, 32]. Two published observational studies systemically investigated the relationships between autoimmune diseases and dementia [33, 34] yielded more positive results than ours. These discrepancies should be interpreted with caution, probably due to the residue confounding and selection bias in different cohorts and study design.

In this study, the risk of dementia was most robust in the early years after hospitalization for the immune-mediated disease. Considering that dementia reflects a late clinical stage in the continuum of disease with long prodromal period when neuropathological changes occur [35], there is possibility that the participants with a dementia diagnosis in the early follow-up years already had neurodegenerative pathology, which could cause the potential reverse causality. Hereto, we performed the sensitivity analyses by excluding the participants with follow-up time less than 5 or 10 years, and the significant associations were only observed for ACD and VD, which could be explained by the much longer disease continuum for AD. Additionally, we reported the sex differences in the associations of immune-mediated diseases with dementia. Our mediation analysis results supported that the associations were partially mediated by peripheral immune cells neutrophils and lymphocytes, and the interaction analyses results indicated that there was an interaction between sex and peripheral immune cells in contributing to the dementia incidence. These findings provide some proof for the statement that sex-specific immune activity could contribute to downstream sex differences in neurodegenerative diseases [36]. In our previous study [8], we have proved the differential role of peripheral innate and adaptive immunity in dementia incidence. The increased neutrophils were associated with higher dementia risk while the elevated lymphocytes were associated with lower dementia risk [8]. The underlined mechanisms of peripheral immunity in dementia remain unclear yet. The assumptions are that these peripheral immune cells arrive at brain in particular disease stages through damaged blood–brain barrier and contribute to abnormal pathological proteins metabolism and inflammatory responses [37–39].

## Limitations

Our study has several limitations. Firstly, the number of dementia cases was small due to the younger, healthier and better-educated background of participants in UKB. In addition, although the positive predictive value (PPV) of ascertaining ACD cases using the UK Biobank data is high, it is lower for AD and VD [40]. Missed dementia diagnoses were still possible and the misclassification of dementia subtype was probable. Secondly, the ascertainment of immune-mediated diseases was based on HESIN data only so we may have missed some diagnoses in other conditions. We also excluded the immune-mediated diseases with less than 500 affected individuals because the dementia incidence was low in some rare immune-mediated diseases. Thirdly, there is no available data of central immune cells such as microglia or data of fluid immune biomarkers such as interleukins in UKB; hence, the mediation analyses were only performed through peripheral immune cells. Fourth, the cohort participants are predominantly European ancestry and of White ethnicity; therefore, some of our findings may not apply to all general populations. Fifth, the control comparators in our study were not totally healthy. We divided the hospital inpatient data recorded participants into two groups according to whether they were diagnosed with immune-mediated disease or not. They could be suffered from some other diseases which may cause the selection bias. Sixth, the participants with immune-mediated diseases may have a more intensive medical follow-up, thus being more likely to be diagnosed with dementia and at earlier stages, which would bias results towards an adverse association between immune-mediated diseases and incident dementia. Finally, although the follow-up time is long and we adjusted for various potential confounders, the possibility of reverse causation remains.

## Conclusions

Our study suggests that immune-mediated diseases were associated with an increased risk of dementia with the risk varying according to type, sex, and timing since immune-mediated diseases. Peripheral immune cells neutrophils and lymphocytes partially mediated such associations. These findings support the importance of immune system dysregulation in dementia pathogenesis. Future studies are needed to confirm our findings in a larger cohort with a longer follow-up period, to improve our understanding of the underlined mechanisms between immune-mediated diseases and dementia, and to explore whether prevention and interventions of immune-mediated diseases reduce the risk of dementia in high-risk populations.

## Supplementary Information

**Additional file 1: Supplementary Table 1.** The list of immune-mediated diseases and their ICD-10 codes used in the study. **Supplementary Table 2.** The list of dementias and their codes used in the study. **Supplementary Table 3.** Baseline characteristics of UKB participants by immune-mediated diseases status. **Supplementary Table 4.** Risk for incident dementia according to the status of any immune-mediated diseases stratified by gender. **Supplementary Table 5.** Sensitivity analysis of the risk for incident dementia according to the status of any immune-mediated diseases. **Supplementary Table 6.** FDR adjusted risk for incident dementia according to the status of individual immune-mediated diseases. **Supplementary Table 7.** Sensitivity analysis of the risk for incident dementia according to the status of individual immune-mediated diseases. **Supplementary Table 8.** The interaction analyses between sex and peripheral immune cells in contributing to the dementia incidence. **Supplementary Figure 1.** Flowchart of the participants selection. **Supplementary Figure 2.** Peripheral immune cells mediation models of the relationship between individual immune-mediated diseases and dementia.

## Data Availability

All data used in this study were accessed from the publicly available UK Biobank Resource under application number 19542. These data cannot be shared with other investigators.

## Abbreviations

Aβ: Amyloid β
ACD: All-cause dementia
AD: Alzheimer’s disease
BMI: Body mass index
CBD: Dementia in corticobasal degeneration
CI: Confidence interval
DLB: Dementia with Lewy bodies
EDTA: Ethylenediaminetetraacetic acid
FDR: False discovery rate
FTD: Frontotemporal dementia
HESIN: Hospital inpatient data
HLA: Human leukocyte antigen
HR: Hazard ratio
PDD: Parkinson’s disease dementia
ICD: International Classification of Diseases
PPV: Positive predictive value
SD: Standard deviation
SNP: Single-nucleotide polymorphisms
TDP-43: Transactive response DNA-binding protein 43 kDa
TGF: Transforming growth factor
TREM2: Triggering receptor expressed on myeloid cells 2
UKB: UK Biobank
VD: Vascular dementia
