# Supplementary material for: Immune-mediated diseases are associated with a higher incidence of dementia: a prospective cohort study of 375,894 individuals
Source: Alzheimers Res Ther. 2022 Sep 13;14:130. doi: 10.1186/s13195-022-01072-x (PMC9472428; doi:10.1186/s13195-022-01072-x)
Supplement: Supplementary file 1 — Additional file 1: Supplementary Table 1. The list of immune-mediated diseases and their ICD-10 codes used in the study. Supplementary Table 2. The list of dementias and their codes used in the study. Supplementary Table 3. Baseline characteristics of UKB participants by immune-mediated diseases status. Supplementary Table 4. Risk for incident dementia according to the status of any immune-mediated diseases stratified by gender. Supplementary Table 5. Sensitivity analysis of the risk for incident dementia according to the status of any immune-mediated diseases. Supplementary Table 6. FDR adjusted risk for incident dementia according to the status of individual immune-mediated diseases. Supplementary Table 7. Sensitivity analysis of the risk for incident dementia according to the status of individual immune-mediated diseases. Supplementary Table 8. The interaction analyses between sex and peripheral immune cells in contributing to the dementia incidence. Supplementary Figure 1. Flowchart of the participants selection. Supplementary Figure 2. Peripheral immune cells mediation models of the relationship between individual immune-mediated diseases and dementia. [file 13195_2022_1072_MOESM1_ESM.pdf]

Longitudinal association of immune-mediated diseases with incident dementia

Supplementary information

Supplementary Table 1. The list of immune-mediated diseases and their ICD-10 codes used in the study

Supplementary Table 2. The list of dementias and their codes used in the study

Supplementary Table 3. Baseline characteristics of UKB participants by immune-mediated diseases status

Supplementary Table 4. Risk for incident dementia according to the status of any immune-mediated diseases stratified by gender

Supplementary Table 5. Sensitivity analysis of the risk for incident dementia according to the status of any immune-mediated diseases

Supplementary Table 6. FDR adjusted risk for incident dementia according to the status of individual immune-mediated diseases

Supplementary Table 7. Sensitivity analysis of the risk for incident dementia according to the status of individual immune-mediated diseases

Supplementary Table 8. The interaction analyses between sex and peripheral immune cells in contributing to the dementia incidence

Supplementary Figure 1. Flowchart of the participants selection

Supplementary Figure 2. Peripheral immune cells mediation models of the relationship between individual immune-mediated diseases and dementia

Supplementary Table 1. The list of immune-mediated diseases and their ICD-10 codes used in the study

	Immune-mediated disease	ICD-10 codes
1	Asthma	J45-J46
2	Rheumatoid arthritis	M05-M06, M08
3	Ulcerative colitis	K51
4	Diabetes mellitus (Type I)	E10
5	Rheumatic fever / rheumatic heart diseases	I00-I02, I05-I09
6	Psoriasis	L40
7	Celiac disease	K90.0
8	Crohn's disease	K50
9	Polymyalgia rheumatica	M35.3
10	Multiple sclerosis	G35
11	Allergic rhinitis	J30.1-J30.4
12	Rheumatism, unspecified	M79.0
13	Psoriatic and enteropathic arthropathies	M07
14	Graves' disease / Autoimmune thyroiditis	E05.0, E06.3
15	Ankylosing spondylitis	M45
16	Necrotizing vasculopathies	M31
17	Sarcoidosis	D86
18	Lichen planus	L43
19	Systemic Lupus erythematosus	L93, M32
20	Idiopathic thrombocytopenic purpura	D69.3

Abbreviations: ICD = International Classification of Diseases.

Supplementary Table 2. The list of dementias and their codes used in the study

Dementias	Code Type	Codes
ACD	ICD-9	290.2, 290.3, 290.4, 291.2, 294.1, 331.0, 331.1, 331.2, 331.5
	ICD-10	A81.0, F00, F00.0, F00.1, F00.2, F00.9, F01, F01.0, F01.1, F01.2, F01.3, F01.8, F01.9, F02, F02.0, F02.1, F02.2, F02.3, F02.4, F02.8, F03, F05.1, F10.6, G30, G30.0, G30.1, G30.8, G30.9, G31.0, G31.1, G31.8, I67.3
	Read V2	1461, A411., A4110, E00.., E000., E001., E0010, E0011, E0012, E0013, E001z, E002., E0020, E0021, E002z, E003., E004., E0040, E0041, E0042, E0043, E004z, E012., E02y1, E041., Eu00., Eu000, Eu001, Eu002, Eu00z, Eu01., Eu010, Eu011, Eu012, Eu013, Eu01y, Eu01z, Eu02., Eu020, Eu021, Eu022, Eu023, Eu024, Eu025, Eu02y, Eu02z, Eu041, Eu106, Eu107, F110., F1100, F1101, F111., F112., F116., F118., F11x2, F11x7, F11x9, F11y2, F21y2, Fyu30, 38C13, 3AE3., 3AE4., 3AE5., 3AE6., 66h.., 6AB.., 8BM02, 8BM50, 8BM60, 8BPa., 8CMe0, 8CMG2, 8CMZ., 8CMZ0, 8CMZ1, 8CMZ2, 8CMZ3, 8CSA., 8Hla., 8IAe0, 8IAe2, 9hD.., 9hD0., 9hD1., 9Ou.., 9Ou1., 9Ou2., 9Ou3., 9Ou4., 9Ou5.
	Read CTV3	.1461, 1461, .E11., .E111, .E112, .E113, .E114, .E115, .E116, .E11Z, .F21Z, .F371, .G78., A411., A4110, E00.., E000., E001., E0010, E0011, E0012, E0013, E001z, E002., E0020, E0021, E002z, E003., E004., E0040, E0041, E0042, E0043, E004z, E012., E02y1, E041., Eu00., Eu000, Eu001, Eu002, Eu00z, Eu01., Eu010, Eu011, Eu012, Eu013, Eu01y, Eu01z, Eu02., Eu020, Eu021, Eu022, Eu023, Eu024, Eu025, Eu02y, Eu02z, Eu041, F110., F1100, F1101, F111., F112., F116., F118., F11x2, F11x7, F11y2, F21y2, Fyu30, Ub1T6, X002m, X002w, X002x, X002y, X002z, X0030, X0031, X0032, X0033, X0034, X0035, X0036, X0037, X0039, X003A, X003B, X003C, X003D, X003E, X003F, X003G, X003H, X003I, X003J, X003l, X003m, X003P, X003R, X003T, X003V, X003W, X003X, X00R2, X00Rk, Xa0lH, Xa0sC, Xa0sE, Xa1GB, Xa25J, Xa3ez, XaA1S, XabVp, XaE74, XaIKB,

		XaIKC, XaKyY, XaOfZ, XE17j, XE1aG, XE1Xs, XE1Xu, XE1Z6, .3AE3, .3AE4, .3AE5, .3AE6, .66h., .6AB., .9hD1, .9Ou., .9Ou1, .9Ou2, .9Ou3, .9Ou4, .9Ou5, 3AE3., 3AE4., 3AE5., 3AE6., 66h.., 6AB.., 8BM02, 8BM50, 8BPa., 8CMe0, 8CMG2, 8CMZ., 8CMZ0, 8CMZ1, 8CMZ2, 8CMZ3, 8CSA., 8IAe0, 8IAe2, 9hD1., 9Ou., 9Ou1., 9Ou2., 9Ou3., 9Ou4., 9Ou5., Xa0fZ, XaaBZ, XaaeA, XaaiW, Xabd2, Xabd3, XabEk, XabEl, XabtQ, XacIx, XacIy, XacIz, XacJ0, XacLx, Xacly, Xaclz, XacM2, Xaefu, XaJBQ, XaJBU, XaJBV, XaJBW, XaJBX, XaJPy, XaLFf, XaLFo, XaLFp, XaMFy, XaMG0, XaMGF, XaMGG, XaMGI, XaMGJ, XaMGK, XaMJC, XaYFR, XaYPX, XaZqJ, XaZWz
AD	ICD-9	331.0
	ICD-10	F00, F00.0, F00.1, F00.2, F00.9, G30, G30.0, G30.1, G30.8, G30.9
	Read V2	Eu00., Eu000, Eu001, Eu002, Eu00z, F110., F1100, F1101, Fyu30
	Read CTV3	.F21Z, Eu00., Eu000, Eu001, Eu002, Eu00z, F110., F1100, F1101, Fyu30, X002x, X002y, X002z, X0030, X0031, X0032, X0033, X003G, XaIKB, XaIKC, XE17j
VD	ICD-9	290.4
	ICD-10	F01, F01.0, F01.1, F01.2, F01.3, F01.8, F01.9, I67.3
	Read V2	E004., E0040, E0041, E0042, E0043, E004z, E012., Eu01., Eu010, Eu011, Eu012, Eu013, Eu01y, Eu01z, F11x2, F21y2
	Read CTV3	.E115, .E116, .G78., E004., E0040, E0041, E0042, E0043, E004z, Eu01., Eu010, Eu011, Eu012, Eu013, Eu01y, Eu01z, F11x2, F21y2, X003R, X003T, X003V, X003W, Xa0lH, XE1Xs

Abbreviations: ACD = All-Cause Dementia, AD = Alzheimer’s Disease, VD = Vascular Dementia, ICD = International Classification of Diseases, Read V2 = Read codes version 2, Read CTV3 = Read codes version 3.

Supplementary Table 3. Baseline characteristics of UKB participants by immune-mediated diseases status (N=375,894)

	None immune-mediated diseases (N=317305)	Any immune-mediated diseases (N=58589)	p-value
Age, y	57.18 (7.80)	58.10 (7.90)	<0.001
Female, n (%)	171171 (53.95%)	34404 (58.72%)	<0.001
ApoE ε4 carriers, n (%)	78039 (28.68%)	14066 (28.28%)	0.071
Higher education, n (%)	113503 (44.84%)	18040 (41.89%)	<0.001
Ethnicity_White, n (%)	299685 (94.96%)	54787 (94.12%)	<0.001
BMI, Kg/m^2	27.52 (4.77)	28.61 (5.53)	<0.001
Townsend deprivation score	-1.30 (3.09)	-0.89 (3.27)	<0.001
Smoking			<0.001
Never, n (%)	168570 (53.44%)	28757 (49.46%)	
Previous, n (%)	112233 (35.58%)	22658 (38.97%)	
Current, n (%)	34626 (10.98%)	6724 (11.57%)	
Alcohol consumption			<0.001
Never, n (%)	13785 (4.36%)	3605 (6.18%)	
Previous, n (%)	11377 (3.60%)	3548 (6.08%)	
Current, n (%)	291119 (92.04%)	51184 (87.74%)	
Incident ACD	4089 (1.29%)	1202 (2.05%)	<0.001
Incident AD	1835 (0.58%)	466 (0.81%)	<0.001
Incident VD	909 (0.29%)	340 (0.59%)	<0.001
Overall health rating	2.14 (0.71)	2.58 (0.81)	<0.001

Data presented as mean (SD) for continuous variables and number (%) for categorical variables. Higher education refers to College/university/other professional qualification.

Abbreviations: ACD = All-Cause Dementia, AD = Alzheimer's Disease, VD = Vascular Dementia, BMI = Body Mass Index.

Supplementary Table 4. Risk for incident dementia according to the status of any immune-mediated diseases stratified by gender

	Model 1 (HR, 95%CI)	Model 2 (HR, 95%CI)	Model 3 (HR, 95%CI)
		ACD	
All	1.27 (1.19-1.35) ***	1.13 (1.03-1.25) **	1.10 (1.00-1.21) *
Female	1.28 (1.17-1.41) ***	1.15 (1.00-1.31) *	1.10 (0.95-1.26)
Male	1.30 (1.18-1.42) ***	1.13 (0.99-1.28)	1.11 (0.97-1.26)
		AD	
All	1.09 (0.98-1.20)	0.91 (0.79-1.07)	0.91 (0.78-1.06)
Female	1.10 (0.96-1.26)	0.88 (0.71-1.09)	0.87 (0.70-1.07)
Male	1.09 (0.93-1.27)	0.96 (0.77-1.19)	0.96 (0.77-1.19)
		VD	
All	1.61 (1.42-1.83) ***	1.52 (1.26-1.83) ***	1.43 (1.19-1.73) **
Female	1.54 (1.27-1.87) ***	1.32 (0.98-1.78)	1.20 (0.88-1.63)
Male	1.76 (1.50-2.08) ***	1.67 (1.32-2.12) ***	1.61 (1.27-2.04) ***

Model 1 unadjusted. Model 2 adjusted for age, sex (for all participants), *ApoE-ε4* and education. Model 3 adjusted for age, sex (for all participants), education, *ApoE-ε4*, ethnicity, BMI, Townsend deprivation score, smoking and alcohol consumption. (*$p < 0.05$, **$p < 0.01$, ***$p < 0.001$)

Abbreviations: ACD = All-Cause Dementia, AD = Alzheimer's Disease, VD = Vascular Dementia, HR = Hazard Ratio, CI = Confidence Interval.

Supplementary Table 5. Sensitivity analyses of the risk for incident dementia according to the status of any immune-mediated diseases by excluding the participants with follow up time less than 5 or 10 years

Follow up time	Model 1 (HR, 95%CI)	Model 2 (HR, 95%CI)	Model 3 (HR, 95%CI)
		ACD	
≥5 years	1.28 (1.20-1.38) ***	1.16 (1.05-1.28) **	1.13 (1.02-1.25) *
≥10 years	1.23 (1.09-1.40) ***	1.22 (1.02-1.45) *	1.17 (0.98-1.40)
		AD	
≥5 years	1.10 (0.99-1.22)	0.94 (0.80-1.11)	0.94 (0.80-1.10)
≥10 years	1.19 (0.99-1.43)	1.13 (0.86-1.47)	1.10 (0.84-1.45)
		VD	
≥5 years	1.63 (1.43-1.87) ***	1.61 (1.32-1.96) ***	1.51 (1.23-1.84) ***
≥10 years	1.65 (1.30-2.09) ***	1.78 (1.26-2.51) **	1.65 (1.16-2.34) **

Model 1 unadjusted. Model 2 adjusted for age, sex, *ApoE-ε4* and education. Model 3 adjusted for age, sex, education, *ApoE-ε4*, ethnicity, BMI, Townsend deprivation score, smoking and alcohol consumption. ($^{*}p < 0.05$, $^{**}p < 0.01$, $^{***}p < 0.001$)

Abbreviations: ACD = All-Cause Dementia, AD = Alzheimer's Disease, VD = Vascular Dementia, HR = Hazard Ratio, CI = Confidence Interval.

Supplementary Table 6. FDR adjusted risk for incident dementia according to the status of individual immune-mediated diseases

Individual immune-mediated diseases	ACD		AD		VD	
	HR (95% CI)	Q	HR (95% CI)	Q	HR (95% CI)	Q
Asthma	1.02 (0.89-1.16)	0.89	0.83 (0.66-1.03)	0.33	1.28 (0.99-1.66)	0.32
Rheumatoid arthritis	1.00 (0.77-1.30)	1.04	1.03 (0.71-1.50)	1.03	0.65 (0.32-1.32)	0.52
Ulcerative colitis	0.80 (0.56-1.16)	0.68	0.37 (0.17-0.83)	0.15	0.52 (0.19-1.39)	0.48
Diabetes mellitus (Type I)	2.49 (1.97-3.15)	6.E-13	2.21 (1.49-3.28)	1.E-03	4.25 (2.90-6.23)	2.E-12
Rheumatic fever / rheumatic heart diseases	1.36 (1.05-1.77)	0.14	1.10 (0.71-1.69)	0.96	2.40 (1.58-3.66)	5.E-04
Psoriasis	1.14 (0.78-1.67)	0.82	0.88 (0.46-1.69)	0.93	2.26 (1.27-4.01)	0.04
Celiac disease	0.83 (0.50-1.39)	0.88	0.95 (0.47-1.90)	0.98	0.57 (0.14-2.27)	0.77
Crohn's disease	0.93 (0.56-1.54)	0.96	0.69 (0.29-1.66)	0.81	0.60 (0.15-2.42)	0.73
Polymyalgia rheumatica	1.00 (0.67-1.49)	1.00	0.71 (0.35-1.43)	0.75	1.29 (0.61-2.72)	0.73
Multiple sclerosis	2.87 (1.92-4.30)	3.E-06	1.11 (0.42-2.97)	1.04	2.50 (0.93-6.71)	0.27
Allergic rhinitis	0.73 (0.36-1.46)	0.83	0.41 (0.10-1.65)	0.60	0.46 (0.06-3.29)	0.74
Rheumatism, unspecified	1.49 (0.82-2.71)	0.62	1.44 (0.60-3.47)	0.76	1.38 (0.34-5.56)	0.87
Psoriatic and enteropathic arthropathies	1.43 (0.74-2.76)	0.71	1.84 (0.76-4.42)	0.59	2.25 (0.72-7.00)	0.46
Graves' disease / Autoimmune thyroiditis	1.15 (0.52-2.57)	0.97	1.28 (0.41-3.98)	1.03	0.99 (0.14-7.06)	0.99

Ankylosing spondylitis	1.28 (0.57-2.84)	0.85	1.04 (0.26-4.18)	1.00	2.03 (0.50-8.13)	0.64
Necrotizing vasculopathies	1.71 (1.03-2.85)	0.19	2.13 (1.06-4.27)	0.23	2.18 (0.82-5.86)	0.40
Sarcoidosis	1.62 (0.81-3.24)	0.70	2.29 (0.95-5.52)	0.32	1.03 (0.14-7.30)	1.09
Lichen planus	0.77 (0.32-1.86)	0.81	0.31 (0.04-2.23)	0.62	0.70 (0.10-5.00)	0.91
Systemic Lupus erythematosus	1.36 (0.61-3.03)	0.91	1.38 (0.44-4.29)	0.96	1.13 (0.16-8.02)	1.07
Idiopathic thrombocytopenic purpura	0.87 (0.33-2.32)	0.92	0.99 (0.25-3.95)	0.98	1.02 (0.14-7.28)	1.03

Adjusted for age, sex, education, *ApoE-ε4*, ethnicity, BMI, Townsend deprivation score, smoking and alcohol consumption. (p values adjusted to control the false discovery rate at 5% using the Benjamini-Hochberg procedure were labeled as Q values)

Abbreviations: ACD = All-Cause Dementia, AD = Alzheimer's Disease, VD = Vascular Dementia, HR = Hazard Ratio, CI = Confidence Interval.

Supplementary Table 7. Sensitivity analyses of the risk for incident dementia according to the status of individual immune-mediated diseases by excluding the participants with follow up time less than 5 or 10 years

Model 1 HR (95% CI)	ACD		AD		VD	
Follow up time	≥5 years	≥10 years	≥5 years	≥10 years	≥5 years	≥10 years
Asthma	1.18 (1.07-1.29) ***	1.18 (1.00-1.38) *	1.00 (0.87-1.16)	1.12 (0.88-1.43)	1.55 (1.30-1.84) ***	1.63 (1.21-2.20) **
Rheumatoid arthritis	1.40 (1.18-1.67) ***	1.47 (1.10-1.96) **	1.45 (1.13-1.87) **	1.60 (1.06-2.41) *	1.31 (0.89-1.93)	1.25 (0.64-2.43)
Ulcerative colitis	0.87 (0.66-1.14)	0.76 (0.46-1.25)	0.61 (0.38-0.98) *	0.52 (0.21-1.24)	0.59 (0.30-1.19)	0.43 (0.11-1.72)
Diabetes mellitus (Type I)	2.83 (2.39-3.35) ***	1.94 (1.39-2.72) ***	2.37 (1.81-3.10) ***	1.33 (0.73-2.43)	4.70 (3.56-6.21) ***	4.52 (2.80-7.32) ***
Rheumatic fever / rheumatic heart diseases	2.00 (1.63-2.45) ***	1.48 (1.00-2.20)	1.41 (0.99-2.02)	1.56 (0.88-2.76)	3.39 (2.43-4.74) ***	2.15 (1.06-4.36) *
Psoriasis	1.49 (1.16-1.92) **	1.72 (1.15-2.58) **	1.26 (0.84-1.89)	1.56 (0.83-2.91)	2.18 (1.40-3.40) ***	2.60 (1.28-5.26) **
Celiac disease	0.91 (0.62-1.34)	0.76 (0.36-1.59)	0.89 (0.51-1.57)	0.92 (0.34-2.46)	0.62 (0.23-1.65)	0.48 (0.07-3.44)
Crohn's disease	0.77 (0.52-1.14)	0.57 (0.27-1.19)	0.57 (0.30-1.11)	0.35 (0.09-1.41)	0.26 (0.07-1.06)	NA
Polymyalgia rheumatica	1.52 (1.11-2.06) **	1.72 (1.06-2.78) *	1.04 (0.60-1.80)	1.10 (0.46-2.66)	1.99 (1.12-3.52) *	1.37 (0.44-4.28)
Multiple sclerosis	1.50 (1.06-2.12) *	0.96 (0.45-2.01)	0.80 (0.40-1.61)	0.89 (0.29-2.76)	1.46 (0.69-3.07)	NA
Allergic rhinitis	0.87 (0.55-1.38)	1.30 (0.67-2.50)	0.52 (0.22-1.25)	1.25 (0.47-3.34)	1.51 (0.72-3.17)	3.27 (1.35-7.94) **
Rheumatism, unspecified	1.36 (0.93-2.00)	1.41 (0.76-2.62)	1.36 (0.77-2.39)	1.23 (0.46-3.28)	1.17 (0.49-2.82)	0.63 (0.09-4.52)
Psoriatic and enteropathic arthropathies	1.23 (0.76-1.98)	1.44 (0.69-3.03)	1.55 (0.83-2.88)	2.21 (0.92-5.34)	0.97 (0.31-3.01)	0.93 (0.13-6.63)
Graves' disease / Autoimmune thyroiditis	1.01 (0.56-1.82)	1.46 (0.60-3.51)	0.98 (0.41-2.36)	0.63 (0.09-4.49)	0.41 (0.06-2.91)	1.34 (0.19-9.52)
Ankylosing spondylitis	1.33 (0.72-2.48)	1.58 (0.59-4.21)	1.72 (0.77-3.82)	1.71 (0.43-6.84)	1.78 (0.57-5.53)	1.77 (0.25-12.59)
Necrotizing vasculopathies	1.95 (1.31-2.92) **	2.02 (1.05-3.90) *	1.74 (0.94-3.25)	1.91 (0.71-5.11)	2.52 (1.20-5.31) *	3.97 (1.48-10.67) **
Sarcoidosis	1.19 (0.68-2.10)	0.59 (0.15-2.38)	0.86 (0.32-2.29)	NA	1.76 (0.66-4.72)	2.68 (0.66-10.77)
Lichen planus	1.40 (0.79-2.47)	1.90 (0.79-4.59)	1.50 (0.67-3.35)	2.48 (0.80-7.73)	1.04 (0.26-4.17)	1.75 (0.24-12.45)
Systemic Lupus erythematosus	1.10 (0.59-2.05)	0.32 (0.04-2.27)	1.65 (0.78-3.46)	0.69 (0.10-4.91)	0.49 (0.07-3.47)	NA
Idiopathic thrombocytopenic purpura	1.19 (0.60-2.39)	1.79 (0.67-4.79)	0.96 (0.31-2.99)	1.95 (0.49-7.81)	1.33 (0.33-5.34)	2.04 (0.29-14.57)
Model 2 HR (95% CI)	ACD		AD		VD	
Follow up time	≥5 years	≥10 years	≥5 years	≥10 years	≥5 years	≥10 years
Asthma	1.06 (0.92-1.22)	1.19 (0.94-1.51)	0.85 (0.68-1.07)	1.19 (0.84-1.69)	1.45 (1.10-1.89) **	1.52 (0.94-2.45)
Rheumatoid arthritis	1.13 (0.87-1.48)	1.39 (0.92-2.09)	1.15 (0.79-1.68)	0.96 (0.47-1.95)	0.89 (0.46-1.73)	1.22 (0.45-3.32)
Ulcerative colitis	0.75 (0.50-1.14)	0.82 (0.41-1.66)	0.35 (0.15-0.84) *	0.23 (0.03-1.62)	0.47 (0.15-1.48)	1.01 (0.25-4.09)
Diabetes mellitus (Type I)	2.45 (1.89-3.16) ***	2.33 (1.47-3.68) ***	2.03 (1.33-3.10) **	1.42 (0.59-3.45)	4.90 (3.26-7.36) ***	5.90 (3.08-11.30) ***
Rheumatic fever / rheumatic heart diseases	1.50 (1.15-1.97) **	1.19 (0.70-2.03)	1.19 (0.77-1.86)	1.30 (0.61-2.75)	2.81 (1.83-4.33) ***	3.18 (1.55-6.55) **
Psoriasis	1.23 (0.83-1.83)	1.19 (0.59-2.40)	1.06 (0.57-1.98)	1.63 (0.67-3.96)	2.35 (1.25-4.40) **	2.17 (0.69-6.85)
Celiac disease	0.94 (0.55-1.58)	0.62 (0.20-1.92)	0.98 (0.47-2.07)	0.88 (0.22-3.53)	0.64 (0.16-2.58)	1.05 (0.15-7.56)
Crohn's disease	1.02 (0.60-1.73)	0.65 (0.21-2.01)	0.78 (0.32-1.87)	0.47 (0.07-3.38)	0.72 (0.18-2.88)	NA
Polymyalgia rheumatica	1.07 (0.71-1.61)	0.82 (0.37-1.83)	0.79 (0.40-1.59)	0.88 (0.28-2.76)	1.07 (0.44-2.60)	NA
Multiple sclerosis	2.50 (1.59-3.92) ***	2.43 (1.09-5.45) *	1.13 (0.42-3.02)	2.72 (0.87-8.51)	2.09 (0.67-6.52)	NA
Allergic rhinitis	0.85 (0.42-1.70)	1.33 (0.50-3.55)	0.46 (0.11-1.83)	1.47 (0.37-5.92)	0.54 (0.08-3.84)	1.73 (0.24-12.40)

Rheumatism, unspecified	1.90 (1.08-3.36) *	2.65 (1.18-5.94) *	1.85 (0.83-4.13)	3.43 (1.27-9.25) *	2.56 (0.82-8.01)	2.73 (0.38-19.70)
Psoriatic and enteropathic arthropathies	1.40 (0.70-2.81)	1.07 (0.27-4.28)	2.24 (1.00-5.01) *	3.60 (1.15-11.26) *	1.78 (0.44-7.15)	NA
Graves' disease / Autoimmune thyroiditis	1.32 (0.59-2.94)	2.42 (0.90-6.48)	1.37 (0.44-4.27)	1.28 (0.18-9.16)	1.14 (0.16-8.10)	3.41 (0.47-24.51)
Ankylosing spondylitis	1.12 (0.47-2.70)	1.38 (0.34-5.52)	0.99 (0.25-3.97)	1.59 (0.22-11.32)	1.15 (0.16-8.17)	NA
Necrotizing vasculopathies	1.77 (1.03-3.06) *	1.83 (0.76-4.42)	1.80 (0.81-4.03)	1.60 (0.4-6.46)	2.62 (0.98-7.02)	3.72 (0.92-15.13)
Sarcoidosis	1.39 (0.62-3.09)	NA	1.51 (0.49-4.69)	NA	1.15 (0.16-8.18)	NA
Lichen planus	0.75 (0.28-1.99)	0.61 (0.09-4.33)	0.37 (0.05-2.61)	NA	0.87 (0.12-6.22)	3.04 (0.42-21.84)
Systemic Lupus erythematosus	1.37 (0.57-3.29)	0.73 (0.10-5.20)	1.61 (0.52-5.02)	NA	1.37 (0.19-9.77)	NA
Idiopathic thrombocytopenic purpura	1.06 (0.40-2.82)	1.38 (0.34-5.53)	1.16 (0.29-4.64)	1.52 (0.21-10.86)	1.25 (0.18-8.89)	NA

Model 3 HR (95% CI)	ACD		AD		VD	
Follow up time	≥5 years	≥10 years	≥5 years	≥10 years	≥5 years	≥10 years
Asthma	1.04 (0.90-1.20)	1.17 (0.92-1.48)	0.84 (0.67-1.05)	1.18 (0.83-1.68)	1.36 (1.03-1.79) *	1.46 (0.91-2.37)
Rheumatoid arthritis	1.09 (0.84-1.43)	1.31 (0.86-1.99)	1.17 (0.80-1.70)	0.95 (0.47-1.93)	0.75 (0.37-1.51)	0.88 (0.28-2.78)
Ulcerative colitis	0.71 (0.47-1.08)	0.71 (0.34-1.50)	0.35 (0.14-0.84) *	0.23 (0.03-1.62)	0.46 (0.15-1.42)	0.99 (0.24-4.02)
Diabetes mellitus (Type I)	2.42 (1.86-3.13) ***	2.23 (1.40-3.54) ***	2.12 (1.39-3.25) ***	1.44 (0.59-3.52)	4.34 (2.87-6.56) ***	5.07 (2.60-9.86) ***
Rheumatic fever / rheumatic heart diseases	1.44 (1.09-1.89) *	1.16 (0.68-1.98)	1.19 (0.76-1.86)	1.27 (0.60-2.7)	2.59 (1.67-4.03) ***	3.02 (1.47-6.23) **
Psoriasis	1.18 (0.79-1.77)	1.04 (0.49-2.19)	0.99 (0.51-1.90)	1.33 (0.49-3.57)	2.22 (1.18-4.16) *	2.05 (0.65-6.47)
Celiac disease	0.92 (0.54-1.56)	0.63 (0.20-1.95)	0.95 (0.45-2.00)	0.89 (0.22-3.56)	0.65 (0.16-2.63)	1.09 (0.15-7.86)
Crohn's disease	1.01 (0.60-1.70)	0.65 (0.21-2.02)	0.78 (0.32-1.88)	0.48 (0.07-3.44)	0.70 (0.17-2.81)	NA
Polymyalgia rheumatica	1.07 (0.71-1.62)	0.82 (0.36-1.83)	0.81 (0.40-1.62)	0.90 (0.29-2.81)	1.05 (0.43-2.54)	NA
Multiple sclerosis	2.55 (1.60-4.07) ***	2.71 (1.21-6.08) *	1.26 (0.47-3.38)	3.09 (0.99-9.7)	2.19 (0.70-6.84)	NA
Allergic rhinitis	0.87 (0.43-1.74)	1.37 (0.51-3.66)	0.47 (0.12-1.88)	1.54 (0.38-6.2)	0.55 (0.08-3.88)	1.75 (0.24-12.53)
Rheumatism, unspecified	1.56 (0.84-2.91)	2.09 (0.86-5.08)	1.62 (0.67-3.91)	3.38 (1.25-9.18) *	1.54 (0.38-6.20)	NA
Psoriatic and enteropathic arthropathies	1.30 (0.62-2.73)	0.55 (0.08-3.94)	2.06 (0.86-4.98)	2.56 (0.64-10.32)	1.73 (0.43-6.95)	NA
Graves' disease / Autoimmune thyroiditis	1.34 (0.60-2.98)	2.50 (0.93-6.69)	1.45 (0.47-4.52)	1.36 (0.19-9.68)	1.11 (0.16-7.90)	3.41 (0.47-24.60)
Ankylosing spondylitis	0.99 (0.37-2.63)	0.74 (0.10-5.28)	0.58 (0.08-4.14)	NA	1.19 (0.17-8.49)	NA
Necrotizing vasculopathies	1.73 (1.00-2.99) *	1.82 (0.75-4.38)	1.80 (0.80-4.02)	1.60 (0.40-6.43)	2.48 (0.93-6.67)	3.72 (0.91-15.11)
Sarcoidosis	1.44 (0.65-3.21)	NA	1.57 (0.50-4.87)	NA	1.18 (0.17-8.42)	NA
Lichen planus	0.73 (0.27-1.95)	0.60 (0.08-4.25)	0.36 (0.05-2.58)	NA	0.83 (0.12-5.91)	2.89 (0.40-20.80)
Systemic Lupus erythematosus	1.31 (0.55-3.17)	0.72 (0.10-5.16)	1.59 (0.51-4.94)	NA	1.29 (0.18-9.20)	NA
Idiopathic thrombocytopenic purpura	1.00 (0.38-2.68)	1.35 (0.34-5.4)	1.10 (0.27-4.41)	1.47 (0.21-10.48)	1.17 (0.16-8.34)	NA

Model 1 unadjusted. Model 2 adjusted for age, sex, *ApoE-ε4* and education. Model 3 adjusted for age, sex, education, *ApoE-ε4*, ethnicity, BMI, Townsend deprivation score, smoking and alcohol consumption. ($^{*}p < 0.05$, $^{**}p < 0.01$, $^{***}p < 0.001$)

Abbreviations: ACD = All-Cause Dementia, AD = Alzheimer's Disease, VD = Vascular Dementia, HR = Hazard Ratio, CI = Confidence Interval.

Supplementary Table 8. The interaction analyses between sex and peripheral immune cells in contributing to the dementia incidence

	Female		Male	
**ACD**	HR (95%CI)	p	HR (95%CI)	p
Neutrophils	1.10 (1.06-1.14)	8.E-07	1.07 (1.04-1.11)	8.E-05
Lymphocytes	0.94 (0.86-1.02)	0.134	0.94 (0.88-1.01)	0.074
**AD**	HR (95%CI)	p	HR (95%CI)	p
Neutrophils	1.06 (1.01-1.13)	0.031	1.03 (0.97-1.09)	0.398
Lymphocytes	0.91 (0.8-1.04)	0.153	0.97 (0.88-1.06)	0.153
**VD**	HR (95%CI)	p	HR (95%CI)	p
Neutrophils	1.13 (1.04-1.23)	0.003	1.09 (1.02-1.17)	0.011
Lymphocytes	1.00 (0.88-1.14)	0.944	0.94 (0.82-1.08)	0.399

Abbreviations: ACD = All-Cause Dementia, AD = Alzheimer's Disease, VD = Vascular Dementia, HR = Hazard Ratio, CI = Confidence Interval.

Supplementary Figure 1. Flowchart of the participants selection

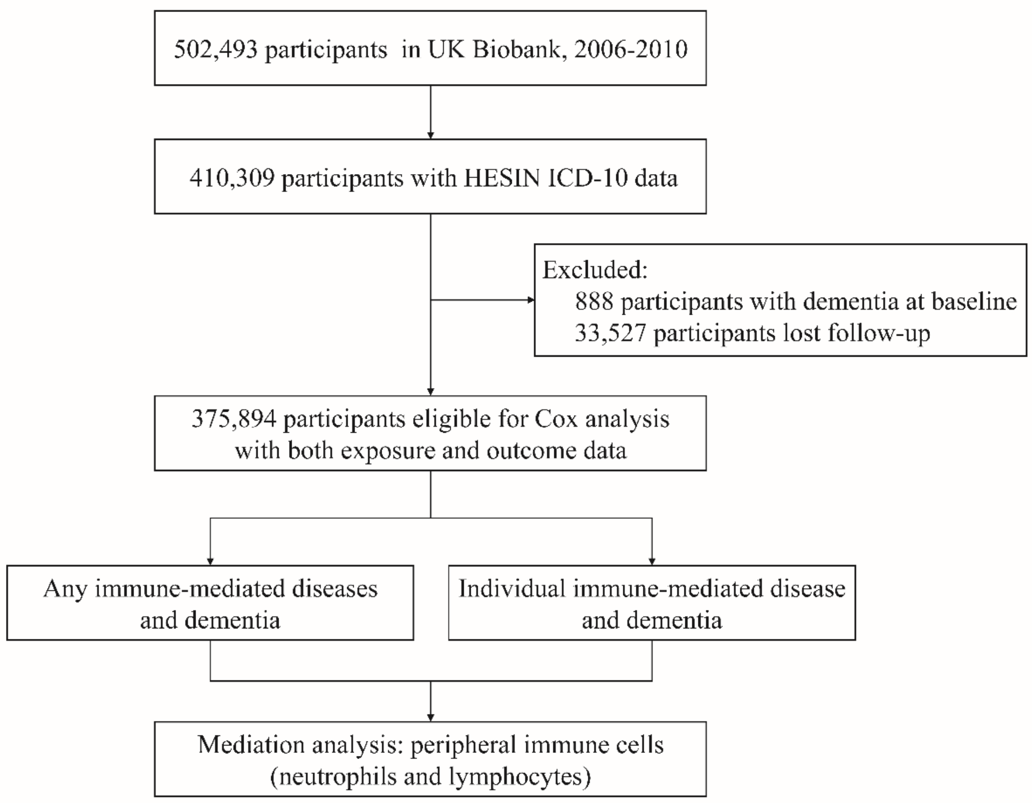

HESIN: Hospital inpatient data.

Supplementary Figure 2. Peripheral immune cells mediation models of the relationship between individual immune-mediated diseases and dementia

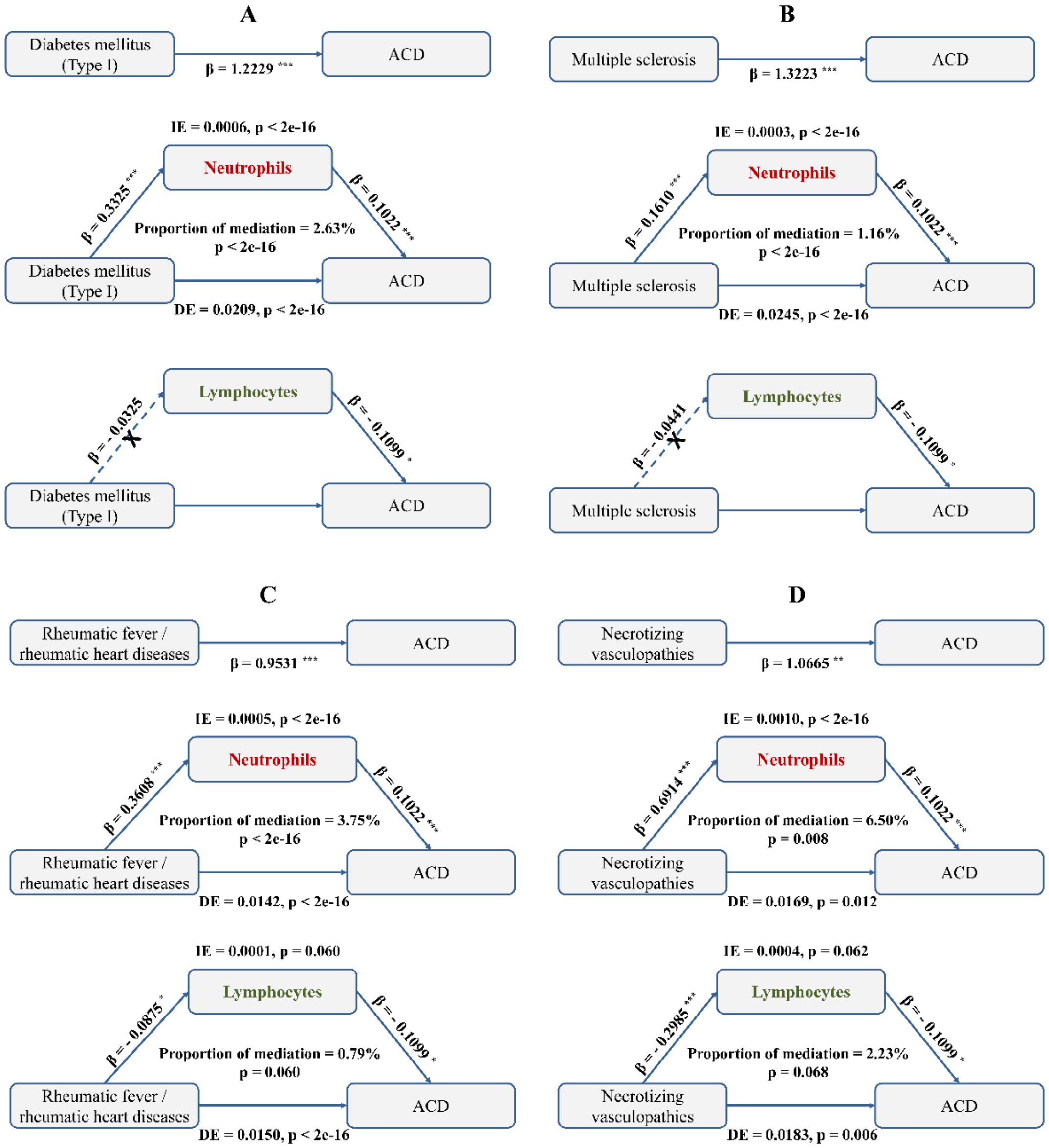

(A) Diabetes mellitus (Type I); (B) Multiple sclerosis; (C) Rheumatic fever / rheumatic heart diseases; (D) Necrotizing vasculopathies. Controlling for age, sex, education, *ApoE-ε4*, ethnicity, BMI, Townsend deprivation score, smoking and alcohol consumption. ($^{*}p < 0.05$, $^{**}p < 0.01$, $^{***}p < 0.001$)

Abbreviations: ACD = All-Cause Dementia, IE = Indirect Effect, DE = Direct Effect.
